# Detection of endometriosis using immunocytochemistry of P450 Aromatase expressions in eutopic endometrial cells obtained from menstrual sloughing: a diagnostic study

**DOI:** 10.1186/s13104-020-05070-w

**Published:** 2020-04-28

**Authors:** Tita Husnitawati Madjid, Jumadi, Raden Tina Dewi Judistiani, Bethy Suryawathy Hernowo, Ahmad Faried

**Affiliations:** 1 Department of Obstetrics and Gynecology, Faculty of Medicine, Universitas Padjadjaran - Dr. Hasan Sadikin Hospital, Bandung, West Java Indonesia; 2 Post Graduate Study of Obstetrics and Gynecology, Faculty of Medicine, Universitas Padjadjaran, Bandung, West Java Indonesia; 3 Department of Public Health, Faculty of Medicine, Universitas Padjadjaran, Bandung, West Java Indonesia; 4 Department of Pathology Anatomy, Faculty of Medicine, Universitas Padjadjaran - Dr. Hasan Sadikin Hospital, Bandung, West Java Indonesia; 5 Oncology and Stem Cell Working Group, Faculty of Medicine, Universitas Padjadjaran, Jl. Eijkman No. 38, Bandung, 40161 West Java Indonesia

**Keywords:** Expression of P450 Aromatase, Endometriosis, Eutopic endometrial cells

## Abstract

**Objective:**

To explore the possibility of a new diagnostic approach of endometriosis based on immunocytochemistry scoring of Aromatase P450 expressions in endometrial cells collected from menstrual sloughing. This is a case control study. Immuncytochemistry scores vs. histopathological examination in one tertiary- and secondary-level hospital in Bandung; two secondary level hospital in Garut and Sumedang, West Java, Indonesia. Thirty-five patients with and without endometriosis were enrolled. All subjects had diagnostic procedures for endometriosis suspicion, with addition menstrual blood samples for cytopathological examination. The specimens were sent for immunocytochemistry assessment of P450 Aromatase expressions (ICAPEC). The previous procedure resulted in cut-off point of histo score (H-score), sensitivity, specificity, (+) and (−) ICAPEC predictive value.

**Results:**

The P450 Aromatase expression in endometrial cells of women with endometriosis was significantly stronger than without one. The cut-off point of H-scores to detect endometriosis was > 4. By this criteria, H-score had 94.6% sensitivity, 90.9% specificity, 92% positive predictive value and 93% negative predictive value. Immunocytochemistry scoring of Aromatase P450 expression in endometrial cells (ICAPEC) derived from menstrual blood specimen was a good candidate as alternatives approach in diagnostic procedure of endometriosis. Application and evaluation in clinical practice would provide the economically benefit in diagnostic procedure.

## Introduction

Endometriosis is endometrium-like tissue outside uterus with similar response towards steroid hormone [1–4]. Endometriosis prevalence is approximately 5–10% of general population and it reached up to 50% in infertile women. The point prevalence of endometriosis (n = 6146, mean age 40.4 ± 8.0 years) was 10.8 per 1000 (95% CI 10.5–11.0). Women aged 40–44 years had the highest prevalence rate of 18.6 per 1000 (95% CI 17.7–19.5). Infertility was documented in 37% of patients. A total of 6045 patients were included in the cohort of newly‐diagnosed endometriosis (mean age 34.0 ± 8.1 years), average annual incidence rate of 7.2 per 10.000 (95% CI 6.5–8.0) [5]. Another issue of endometriosis is the disturbance the quality of life and fertility caused by accession, change of peritoneum function, hormonal, immunology and high relapse occurrence [6].

The growth of endometriosis is hormone dependent; evidenced by estrogen and progesterone receptor in endometriosis epistle and stromal [7]. It is reasonable to address the cellular change assessment due to hormonal influences. Recently, aromatase enzyme occurrence in endometriosis implantation which affects estrogen biosynthesis which takes part in endometriosis development. Estrogen is recognized as endometrial mitogen; therefore, the existence of estrogen production in ectopic endometrial itself is able to explain the failure of hormonal medication to some patients with endometriosis. Moreover, the appearance of aromatase in endometriosis implantation supports the assumption that the production of local estrogen will develop the growth of endometriosis [6].

Aromatase was discovered by Noble, Simpson et al. [8] in eutopic endometrium and endometriosis implantation in patients with endometriosis, whereas normal endometrium and peritoneum in women without endometriosis do not show the appearance of aromatase. It was also biochemically proven that eutopic endometrium and endometriosis implantation in patients with endometriosis is different from women without endometriosis. Aromatase can be observed using immunohistochemistry technique and is not found in endometrium of cervical carcinoma patients without other gynecologic diseases; it is evident that this examination is very sensitive and specific. Our previous study shown endometrium cells that survive in menstrual blood can be isolated and analyzed for several proteins using immunocytochemistry that conducted based on histo score (H-score) by intensity and distribution of colored cells which is called semi quantitative examination.

The finding of aromatase enzyme in eutopic endometrium in women proven to suffer with endometriosis brought up the question whether aromatase activity can be found and assessed through the examination of menstrual blood of patients with endometriosis and whether there is significant difference in the appearance of aromatase between patients with and without endometriosis; we can conclude a theme of the main problem as follows: The growth of endometriosis depends on hormone especially estrogen as endometrium mitogen. Estrogen is not only produced in ovary but also produced locally in endometriosis implantation which plays a role in the growth and development of endometriosis and assisted with the occurrence of aromatase activity [9]. Aromatase is also found not only in endometriosis implantation but also in eutopic endometrium with endometriosis [8, 10].

The diagnosing endometriosis is by laparoscopic surgery. However, it still faces problems since it is invasive and not all patients are willing to do it [1]. Other non-invasive methods have been propose such as P450 aromatase in endometrium that survive in menstrual blood of endometriosis patients. Our aim was to analyze the appearance of P450 aromatase based on the intensity and distribution in menstrual blood between patients with endometriosis and control. We hypothesized that there were significant differences in aromatase expression in menstrual blood in endometriosis patient compared to controls. If it expression has a diagnostic or predictive value, it can be used to detect endometriosis non-invasively in the early stages, so that the management of endometriosis can be done better. Furthermore, better management can also prevent further complications.

## Main text

### Materials and methods

This is a case–control study that measure the P450 Aromatase in patient’s menstrual blood with endometriosis by using laparoscopic or laparotomy surgery, confirmed with histopathology at Department of Obstetrics and Gynecology, Dr. Hasan Sadikin Hospital (RSHS), Bandung, Public Hospital in Sumedang and Ujung Berung, also from Dr. Slamet Hospital, Garut. Sample size was based on a 95% confidence level, by setting the sensitivity of the P450 aromatase examination results in estimating the endometriosis event by 80% and choosing a precision of 15% from the sample size formula and considering the mild endometriosis incidence rate according to the literature is 30%, then the number of n are: 92, plus 10%, so that the minimum sample reaches 100 patients. Patient selection was carried out by team (obstetrics-gynecology specialists and residents); patient will undergo diagnostic laparoscopic or definitive surgery both laparoscopic and laparotomy. Patients who underwent Pomeroy sterilization had their menstrual blood collected prior the surgery, in the first days of menstruation out a lot (day I–II) to P450 aromatase immunocytochemistry.

Inclusion criteria: histopathological results of endometriosis; non-endometriosis included as control group. The exclusion criteria: pregnant women, on hormone treatment, at least 3 months, except GnRH after 9 months of the last administration; other gynecological diseases (infections, malignancies), patients who have received hormone therapy before menstrual blood sampling and surgery, whose data were incomplete and samples are less representative were also excluded. In the implementation, there were 63 (42.28%) cases of endometriosis, while 86 (57.72%) were classified as non-endometriosis. All representative sample evaluation: 37 endometriosis vs 33 control groups.

#### Immunocytochemistry

One ml menstrual blood was collected, one per subject on the 1–2 day of the cycle and into a sterile container; were then centrifuged 15 min. Immunocytochemistry smears were made on the object’s glass. They were soaked in xylol and ethanol I, II and III, as well as in alcohol 70, 80 and 90% for 5 min each, then washed with distilled water. The antigen unmasking retrieval procedure was done for 3 × 5 min, then cooled at room temperature 15 min; washed with phosphate buffer saline (PBS). Then incubated with 0.3% H_2_O_2_ in methanol 10 min; washed with PBS. The smears were added the blocking reagents and were incubated 5–10 min. Then given primary antibodies, incubated 60 min; washed PBS 3x. Secondary antibodies were then added, incubated 10 min; washed PBS 3x. Later on, streptavidin were used and were incubated 10 min; washed PBS 3x. Chromogen was dropped and incubated 10 min; washed with running water 5 min. The smears were Incubated in mayer hematoxylin for 2 min; washed with running water after. The last steps of the procedure were to dehydrate alcohol 70, 80, 90% for 3 min and soak the smears into xylol for 3 min, then mounting.

#### Assessment of immunocytochemistry

Histopathological endometriosis criteria: found endometrial gland epithelial cells and endometrial stromal in the tissue examined. The criteria for viable cells in this study were: there were cells or groups of stromal cells in their entirety in menstrual blood smear preparations. Generally, most cells will soon die when exposed to sunlight. Menstrual blood contains many proteolytic enzymes that are released from lysosomes which break due to a decrease in steroid hormones before menstruation, so that the menstrual blood cell component is easier to experience lysis. Lymphocyte cells around the group of stromal cells that are also stained with dark brown are used as positive controls.

#### Statistical analysis

Data normality were assessed. Comparison of continuous variables were done using unpaired t-test if normally distributed; otherwise, Mann–Whitney U test was used. Categorical variables were compared using Chi square test. To determine the cut-off value of aromatase P450 expression in detecting endometriosis, ROC curve was used. *p* value < 0.05 were considered significant.

### Results

#### Patient characteristics and expression of P450 Aromatase in eutopic endometrial cells

There were 5 samples that were not evaluable due to the lack of endometrium cells or no cell at all. The samples that quantified as 37 endometriosis and 33 non-endometriosis cases. They were on the stages of III and IV (severe). Based on the characteristics of the subjects in Table 1, all variables of age, job, marital status and body mass index do not show significant differences between both groups. Based on the homogeneity of data above, both groups were legible to be compared. Table 2 shown that endometrium cells in menstrual blood of patients with endometriosis displays the appearance of P450 Aromatase with strong, medium, weak and negative intensity by 25 (67.6%), 12 (32.4%), 0 and 0 cases while in control group there were 3 (9.1%), 9 (27%), 18 (54.5%) and 3 (9.1%) cases.

**Table 1:** Comparison of study subjects’ characteristics

Characteristics	Group		*p* value
	Endometriosis (n = 37)	Non endometriosis (n = 33)	
Age (in years)			
Mean (SD)	32.9 (6.9)	31.8 (9.0)	0.576*
Median	34.0	31.0	
Min–max	20–49	17–54	
Occupation			
Housewife	23	21	0.618**
Employed	14	12	
Marital status			
Married	34	28	0.355**
Single	3	5	
Social economy			
a. Upper	0	4	0.060**
b. Middle	17	10	
c. Low	20	19	
BMI (kg/m^2^)			
Mean (SD)	22.8 (3.5)	22.7 (3.8)	0.882*

* t test, ** x^2^ tst

**Tabel 2:** Subjects distribution based on H-scoring of ICAPEC and P450 Aromatase endometrium cells in menstrual blood of patients with endometriosis and control

	Group				OR (95% CI)	*p* value
	Endometriosis		Control			
	n	%	n	%		
H scoring						
Negative	0	0	3	9.1	–	0.001*
Weak	0	0	18	54.5	–	
Medium	12	32.4	9	27	1.0	
Strong	25	67.6	3	9.1	6.25 (1.2–36.2)	
P450 Aromatase (%)						0.001*
Negative	0	0	3	9.1	–	
< 20	7	0	15	48.5	–	
20–50	17	18.9	11	33.3	1.0	
50–80	13	45.9	2	6.1	13.36 (1.93–118.02)	
> 80	13	35.1	2	6.1	10.21 (1.43–92.35)	

*Chi square test, H-scoring: histophatology score, ICAPEC: immunocytopathological expression of P450 aromatase in endometrial cells

**Fig. 1:**
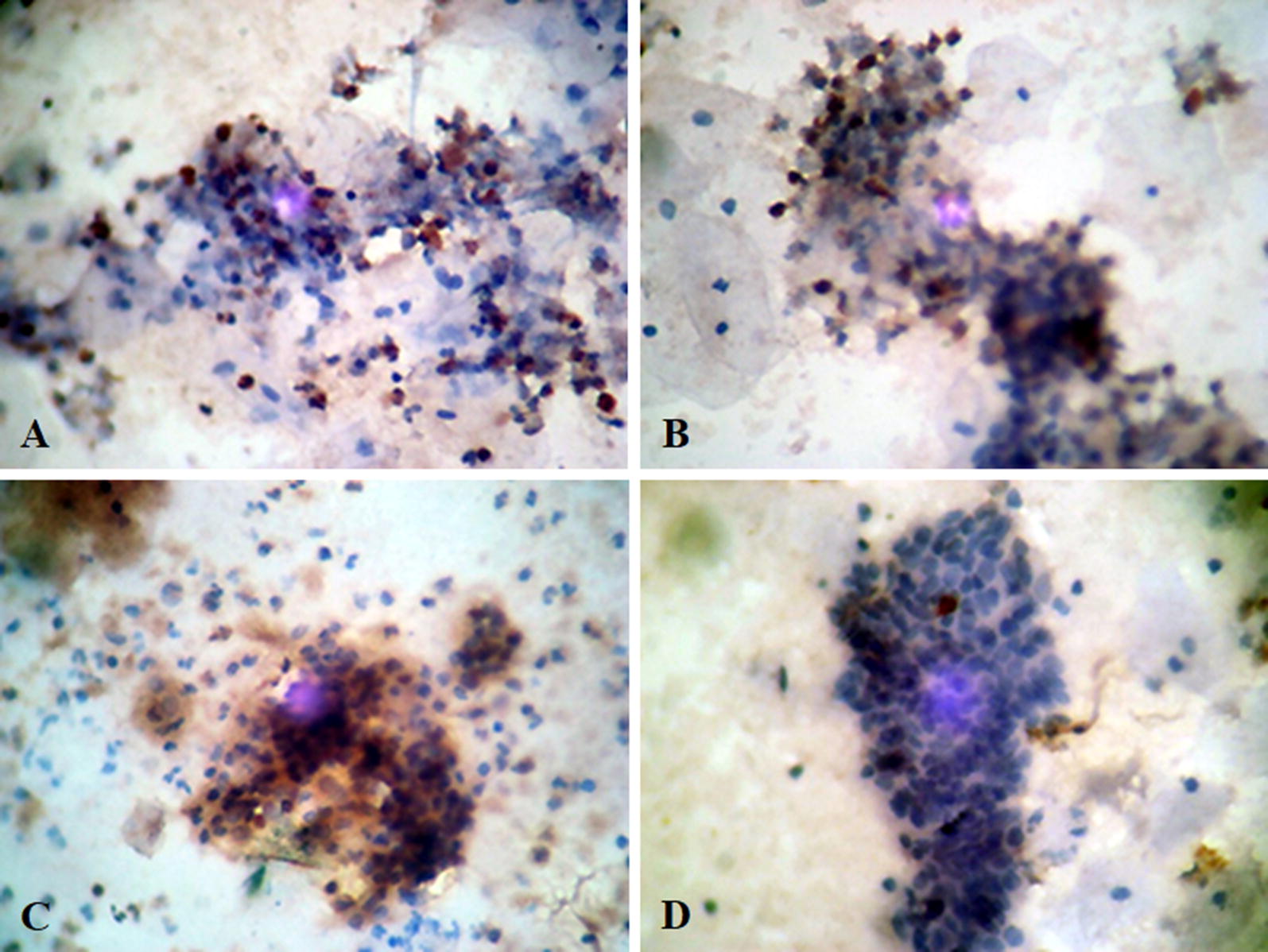
Endometrial cells with endometriosis that have been successfully isolated from menstrual blood can be stained by using immunocytochemical techniques to examine the appearance of P450 aromatase. **a**–**d** Appear consecutively in the appearance of P450 aromatase with cell distribution, i.e. dark brown cells of 20–50%, 50–80%, > 80% and cells that do not show aromatase display P450

Endometrial cells in menstrual blood shown different Aromatase P450 expression intensities. Cell distribution was assessed based on the number of cells stained in each field of view are shown (Fig. 1). Table 2 shown the distribution of the P450 Aromatase expression in patients menstrual blood with endometriosis by > 80%, 50–80%, 20–50%, < 20% and negative from each of 13 (35.1%), 17 (45.9%), 7 (18.9%), 0 and 0 cases while in control group, each group shows 2 (9.1%), 2 (9.1%), 11 (33.3%), 15 (48.5%) and 3 (9.1%) cases, respectively. The difference in the appearance of P450 Aromatase (H-Score) between the two groups is significant. Receiver operating characteristics (ROC) Curve is used to decide the cut off point of the appearance of P450 as follows: with cut off point > 4, it is found that the sensitivity is 94.6% and the specificity is 90.9% (Additional file 1). Using 2 × 2 templates, it is found that positive predictive value is 92% and negative predictive value is 93%. With a positive predictive value of 92%, it can be assumed that when the Aromatase found in endometrium cells in menstrual blood, then the chance of a woman to suffer from endometriosis is 92%. With a negative predictive value of 93%, it can be assumed that when the Aromatase not found in endometrium cells in menstrual blood, then the chance for a woman not to suffer endometriosis is 93%.

### Discussion

The analysis of the characteristics of the research subjects covers age, job, marital status, social and economy status, and body mass index, which shows that there is no significant difference (p = 0.576) in both groups; therefore, they are able to be compared. It is shown that the P450 Aromatase expression in menstrual blood of endometriosis patients differs significantly compared to control. In patients with endometriosis, the P450 Aromatase expression shows cases with medium intensity of 32.4% and strong intensity of 67.6% (OR 6.25; 95% CI 1.21–36.25) and cases with negative and weak intensity were not found (Table 2).

The distribution of P450 Aromatase in endometriosis patients also differs significantly vs. control with the distribution of 20–50% in 18.9% cases, 50–80% in 45.9% cases (OR 13.36; 95% CI 1.93–118.02) and > 80% in 35.1% cases (OR 10.21; 95% CI 1.43–92.35); case with negative distribution and < 20% was not found. In control shown different expression and 9.1% cases with negative result; 3.0% cases were found negative result with the distribution > 80% (Table 2). Our result shown 91% sensitivity, 100% specificity, 100% positive- and 72% negative-prediction value. Another study also acquired a result of 100% sensitivity, 75% specificity, 86.9% positive- and 100% negative-prediction value. Our study shown 94.6% sensitivity, 90.6% sensitivity, 92% positive- and 93% negative-prediction value. The findings of the P450 Aromatase expression that obtained from menstrual blood by conducting immunocytochemistry examination and done non-invasively can be used to support the diagnosis for the establishment of endometriosis diagnosis.

Until recently, the main causes of endometriosis are still unknown. Retrograde menstruation occurs in 76–90% women. The prevalence of endometriosis which is far lower as 6.2–8.2% shows other factors in determining the susceptibility towards endometriosis. The response of aberrance immune that shows the inadequacy of menstrual reflux debris is one of the possible factors that tempted the occurrence of endometriosis other than genetic factor.

### Conclusion

There were differences in the intensity and distribution of P450 Aromatase in patient menstrual blood with and without endometriosis. ICAPEC derived from menstrual blood specimen was a good candidate for alternative diagnostic procedure of endometriosis. Application and evaluation in clinical setting can provide the economical benefit of immunocytochemistry in diagnostic procedure.

## Limitation of the study

The limitation to this study is that the subjects do not represent all; only stage III and IV (severe).

## Supplementary information

**Additional file 1. Figure S1.** ROC curve cut off point of the appearance of P450 Aromatase.

## Data Availability

Authors declare that the data will not be shared since they are patients’ confidentiality.

## Abbreviations

ICAPEC: Immunocytochemistry assessment of P450 Aromatase expressions
H-score: Histo score
RSHS: Dr. Hasan Sadikin Hospital
PBS: Phosphate buffer saline
ROC: Receiver operating characteristics
