# Supplementary figures and images for: Detection of endometriosis using immunocytochemistry of P450 Aromatase expressions in eutopic endometrial cells obtained from menstrual sloughing: a diagnostic study
Source: BMC Res Notes. 2020 Apr 28;13:233. doi: 10.1186/s13104-020-05070-w (PMC7189717; doi:10.1186/s13104-020-05070-w)

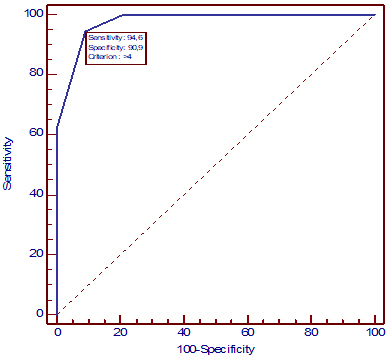

Supplement: Supplementary file 1 — Additional file 1. Figure S1. ROC curve cut off point of the appearance of P450 Aromatase. [file 13104_2020_5070_MOESM1_ESM.jpg]
